# Association Between the Use of Wearable Devices and Physical Activity Among US Adults With Depression and Anxiety: Evidence From the 2019 and 2020 Health Information National Trends Survey

**DOI:** 10.7759/cureus.39521

**Published:** 2023-05-26

**Authors:** Okelue E Okobi, Temitope O Sobayo, Abimbola E Arisoyin, Damilola A Adeyemo, Kehinde T Olaleye, Chika O Nelson, Ibilola A Sanusi, Mujeeb A Salawu, Agatha O Akinsete, Erhieyovbe Emore, Chidalu N Ibeneme, Victor A Odoma, Adeniyi K Busari, Emeka Okobi

**Affiliations:** 1 Family Medicine, Medficient Health Systems, Laurel, USA; 2 Family Medicine, Lakeside Medical Center, Belle Glade, USA; 3 Psychiatry and Behavioral Sciences, All Saints University School of Medicine, Roseau, DMA; 4 Internal Medicine, University of Lagos, Lagos, NGA; 5 Medicine, Texas Agricultural and Mechanical (A&M) University, Corpus Christi, USA; 6 Neurological Sciences, Tampa General Hospital, Tampa, USA; 7 Epidemiology and Public Health, Lagos State Ministry of Health, Lagos, NGA; 8 Internal Medicine, University of Texas at Houston, Houston, USA; 9 Medicine and Surgery, College Of Health Sciences, University Of Ilorin, Ilorin, NGA; 10 Internal Medicine and Psychiatry, Houston Health Department, Houston, USA; 11 Medicine and Surgery, Obafemi Awolowo University, Ile Ife, NGA; 12 Biomedical Sciences, University of Lagos, Lagos, NGA; 13 Family Medicine, Abia State University, Uturu, NGA; 14 Cardiology and Oncology, Indiana University health, Bloomington, USA; 15 General Practice, Emory Rollins School of Public Health, Atlanta, USA; 16 Dentistry, Ahmadu Bello University Teaching Hospital, Abuja, NGA

**Keywords:** mental disorder, anxiety, depression, health promotion, physical activity, wearable devices

## Abstract

Objective

The objective of this study was to examine the relationship between wearable device (WD) use and physical activity (PA) levels among US adults with self-reported depression and anxiety.

Methods

Data were pooled from 2026 adults who self-reported depression and anxiety from the 2019 and 2020 Health Information National Trends Survey. The explanatory variable was WD use, and the outcomes were weekly PA levels and resistance strength training. Logistic regression was conducted to investigate the association between WD and PA parameters.

Results

About 33% of adults with self-reported depression/anxiety reported WD use. Only 32.5% and 34.2% of the population reported meeting the weekly recommended levels of physical activity (≥150 minutes/week) and strength and resistance exercise (≥2 times weekly), respectively. In adjusted analyses, the use of WD was not associated with meeting the national weekly recommendation for physical activity (OR 1.38, 95% CI (0.94, 2.04); p=0.10) or resistance strength training (OR 1.31, 95% CI (0.82, 2.08); p=0.26). Further exploratory analysis also showed that physical activity levels did not differ with the frequency of WD use.

Conclusion

Despite the popularity of WD use among people with mental disorders, we found that use of WD was not associated with increased physical activity measures, suggesting that although there is a promise for these tools to augment mental health, their real-world effectiveness in promoting physical activity in people with mental disorders remains to be proven.

## Introduction

Mental disorders such as depression and anxiety remain a significant cause of morbidity and mortality, affecting millions of people every year. According to the World Health Organization (WHO), approximately 280 million people worldwide have depression, and another 301 million have anxiety disorders, making them the two most common mental health disorders globally [1]. Concerningly, recent data suggest that the prevalence of these disorders may have increased due to the coronavirus pandemic [2].

Mental disorders are associated with higher morbidity, and people with these conditions have a lower quality of life and experience earlier mortality than the general population [3-4]. It is widely acknowledged that this excess mortality is due to the greater burden of chronic diseases, poor physical health and poor cardiovascular health among individuals with mental disorders [3-5].

Physical activity has demonstrated benefits in improving morbidity and mortality in the general population [6]. The benefits of physical activity also extend to individuals with depression and anxiety disorders. Multiple observational studies, systematic reviews, and meta-analyses have consistently reported that across the spectrum of mental disorders, engagement in physical activity significantly reduces the morbidity and mortality associated with mental illness, a growing body of literature indicates that physical activity also confers protection against the development of some depressive and anxiety disorders [7-9]. As a result, leading health organizations have suggested that physical activity and exercise be considered effective adjunctive treatments for mental disorders such as anxiety and depression [10-11].

Despite the known benefits of physical activity, many people with depression and anxiety disorders do not engage in regular physical activity and accumulate a substantial amount of sedentary time [12-13]. Lack of motivation, low self-esteem, and poor self-efficacy are some of the reasons for low physical activity among people with mental disorders. Given the potential benefits of physical activity on mental health outcomes, it is crucial that innovative approaches and strategies are implemented to encourage and support physical activity in people with mental disorders.

Wearable devices, such as fitness trackers, have been gaining popularity as tools to monitor physical activity levels and motivate individuals to engage in physical activity [14]. These tools are convenient, user-friendly, and can provide real-time feedback on the wearer's physical activity level. Previous studies have shown that these devices are acceptable and feasible in promoting physical activity in the mentally ill population [15-16]. However, the efficacy of these tools in improving physical activity in people with mental disorders. Past works have been limited to controlled settings, smaller populations, and recruited participants who were already motivated to lose weight. One cross-sectional study on the real-world potential for wearable devices to increase physical activity in people with depression and anxiety disorders found benefits for these tools in people who had intentions to lose weight [17]. As the use of wearable devices continues to grow, we sought to further extend the literature on the role of wearable devices in promoting physical activity in mental health contexts. Therefore, we conducted a cross-sectional study to evaluate this association, drawing from a nationally representative sample of US adults with self-reported depression and anxiety disorders. We also examined if the frequency of wearable device use is associated with physical activity outcomes.

## Materials and methods

Procedures

In this study, data was collected from the Health Information National Trends Survey (HINTS), which is a household interview survey conducted by the National Cancer Institute (NCI). HINTS is designed to be nationally representative and targets non-institutionalized adults aged 18 years or older residing in the United States. Since 2003, the NCI has administered the survey every few years [18]. HINTS primarily collates information related to cancer diagnosis, treatment, and prevention. It also collects data on several other topics from the general population, including lifestyle behaviors as well as the utilization of health communication systems between health providers and patients. The data used in this study is from cycles three and four of the fifth iteration of the HINTS, which were collected from January 22 to April 30, 2019, and February 24 to June 15, 2020, respectively.

Detailed information regarding the methodology employed for data sampling, collection, and weighting of both HINTS datasets has been previously published [19]. The HINTS 5 surveys utilized a two-stage, stratified random sampling method. In the first stage, non-vacant residential addresses were selected from the Marketing Systems Group (MSG). The second stage involved the selection of one adult from each household to participate in the survey, which was done using the "Next Birthday" method. The residential addresses database was then grouped into two: The "low-minority strata" (areas with <34% Hispanics or African Americans) and the 'high-minority strata' (areas with ≥34% Hispanics or African Americans). This stratification was done to increase the precision of estimates for minority subpopulations. The survey respondents were then weighted to reflect selection probabilities and to provide a nationally representative sample in terms of gender, age, marital status, educational attainment, ethnicity, race, and census region. Apart from the full-sample weight, 50 replicate weights were assigned to each adult in the survey. These replicate weights were utilized to estimate the standard error of the obtained HINTS data estimates, using the delete-one jackknife (JK1) replication method [20].

These HINTS study participants provided written informed consent prior to their participation. HINTS fifth edition cycles three and four received approval from the Westat Institutional Review Board (IRB) and were classified as exempt from review by the US National Institutes of Health Office of Human Subjects Research Protections, as the data was de-identified. This study adhered to the Strengthening the Reporting of Observational Studies in Epidemiology (STROBE) reporting guideline recommendations [21].

Study design and participants

A cross-sectional design was used to evaluate participant responses from both HINTS five cycle three (H5C3) and HINTS five cycle four (H5C4). The combined H5C3 and H5C4 datasets contained responses from 9303 survey respondents: 5438 and 3865 respondents from cycles three and four, respectively. As in previous studies [17,22], participants with depression and/or anxiety were ascertained from responses to the survey question: "Has a doctor or other health professional ever told you that you had depression or anxiety disorder?". Those respondents who answered yes were classified as having depression and/or anxiety and included in the study. The overall household response rate was 30.3% in H5C3 and 37% in H5C4. Of note, participants surveyed at each cycle were different, and consequently this was not a repeated measures design.

Measures

The main exposure measure of this study was self-reported use of WD within the past 12 months preceding the survey. To assess exposure, respondents were first asked: "In the past 12 months, have you used an electronic wearable device to monitor or track your health or activity? For example, a Fitbit, Apple Watch, or Garmin Vivofit?". Response options were "yes" or "no", and those who answered yes were then classified as WD users. We also sought to explore the association between frequency of WD use and physical activity levels. Frequency of WD use was derived from the survey question; In the past month, how often did you use a wearable device to track your health? Response options ranged from "every day", "almost every day", "one to two times per week", "less than once per week" to "I did not use a wearable device in the past month". For this study, given the small numbers of affirmative responses to the frequency of WD utilization question, we reclassified frequency of WD use into three categories. Respondents who answered "no" to the initial question regarding use of wearable devices were classified as non-users of WD. Respondents who indicated one to two times per week or less to the frequency of WD use question were classified as infrequent users, and those who reported >2 times per week of WD use were classified as frequent users.

Outcome measures

We studied two physical activity related outcomes: weekly physical activity, and strength training. Both outcomes were ascertained using questions derived from the HINTS survey. Specifically, weekly physical activity was determined using the following two survey questions: 1) "In a typical week how many days do you do any physical activity or exercise of at least moderate intensity, such as brisk walking, bicycling at a regular pace, and swimming at a regular pace?", where response options ranged from none to seven days per week; and 2) "On the days that you do any physical activity or exercise of at least moderate intensity how long do you do these activities?", where response options ranged from minutes and hours. Responses to these two questions (the days of having physical activity and the time of each day) were multiplied to obtain the weekly average time for physical activities for each respondent. Next, based on the Federal Physical Activity Guidelines for Americans and World Health Organization (WHO) recommendations and guidelines for physical activity levels [23-24], we then reclassified the respondents into two groups for physical activity levels, that is, whether the subject met physical activity recommendations (≥150 minutes per week) or did not meet the physical activity recommendations (<150 minutes per week).

Next, weekly level of strength training was determined from the survey question: "In a typical week, outside of your job or work around the house, how many days do you do leisure-time physical activities specifically designed to strengthen your muscles?", where response options ranged from one day per week to seven days per week. Strength training was dichotomized based on the Center for Disease Control (CDC) national recommendations of resistance and strength exercise of ≥2 times/week into inadequate (<2 times/week) and adequate (≥2 times/week) [25].

Covariates

Covariates included in the present study were age, gender, race, educational level, household income, insurance status, comorbidities, access to a regular health care provider, body mass index, and geographical residence. Age was grouped into four categories: 18 to 34 years, 35 to 49 years, 50 to 64 years, and 65 years or older. Race/ethnicity was categorized as non-Hispanic White, non-Hispanic Black, Hispanic, and others; and educational level was grouped into four categories: high school graduate or lower, some college, college graduate, and postgraduate. Household annual income was categorized as less than $20,000, $20,000 to $34,999, $35,000 to $49,999, $50,000 to $74 999, and $75,000 or more. Respondents were classified as having a comorbidity if they had one or more of the following conditions: diabetes mellitus, hypertension, heart disease, and lung disease. Rural/urban residence was defined using the Rural-Urban Continuum (RUC) Code. Codes one to three were urban, representing commuting patterns to metro counties with populations of ≥250,000. Codes four to nine were rural, representing non-metro counties with populations of 2500 to 20,000.

Statistical analysis

We first conducted basic descriptive statistics for the entire study sample and by WD status. Weighted percentages were presented. Chi-squared tests were used to compare WD use across respondents' sociodemographic and health-related characteristics. Next, two separate multivariable logistic regression models were estimated to investigate the relationship between WD use and physical activity parameters (weekly physical activity, and weekly strength/exercise resistance training) in the entire study population adjusting age, gender, race, educational level, household income, insurance status, comorbidities, access to a regular health care provider, body mass index, and geographical residence. Next, we performed further exploratory analysis using multivariable logistic regression models to evaluate the association between the frequency of WD use with weekly physical activity and strength/exercise resistance training adjusting for the same covariates.

We performed all statistical analyses using the "svy" command in Stata 17.0 statistical software (StataCorp LP, College Station, Texas). Final person weights and jackknife replicate weights provided within the H5C3 and H5C4 datasets were used to estimate national-level values and standard errors of estimates respectively. All tests were two-sided, and p-values of <0.05 were considered statistically significant.

## Results

Of the complete sample, we identified a total of 2,026 adults who self-reported depression and/or anxiety and provided responses regarding the use of WD and thus were included in the final analyses. In the full sample, 61.5% were females, about 43.3% were aged 50 years or more, 27.0% had completed college degrees, 70.6% were non-Hispanic Whites, 33.5% earned above $75,000 dollars annually and 53.3% had at least one comorbidity.

A total of 541 participants (weighted percentage; 30.3%) reported a past 12-month use of WD. Among those with depression and/or anxiety who reported using WD, about 30% reported infrequent use, and 70% endorsed frequent use within the past month. Approximately 32.5% of the population reported meeting the nationally recommended levels of weekly physical activity (≥150 min/week), and about 34.2% reported adherence to weekly resistance and strength exercise (≥2 times/week).

Overall, in bivariate analyses (Table 1), those with depression and/or anxiety who reported WD use in the last year were more likely to be women (35.9%) vs. men (22.8%), younger adults (46.5% were aged 18-34 years) vs. older adults (10.7% were aged 65 years or more), post-graduates (45.4%) vs. people with less than a high school education (17.2%), people with high income (40.4% were from households with annual income ≥$75,000) vs. people with lower income (22.7% were from households with annual income ≤$20,000), and people with no comorbidity (37.8%) vs. people with two or more comorbidities (15.9%). There was no significant difference in WD usage by race, insurance status, access to a regular provider, and geographical residence. However, non-obese individuals (33.8% vs. 24.7%), those who met the national recommended guidelines for weekly physical activity (39.0% vs. 26.0%), and strength training (36.1% vs. 27.3%) were more likely to use WD. Full details on the distribution of the sociodemographic characteristics of the study population by WD use are shown in Table 1.

**Table 1:** Sociodemographic characteristics and physical activity status of the study participants WD - wearable device

Demographic variables	Total, % (n=2026)	No use of WD, % (n=1485)	Use of WD, % (n= 541)	p-value
Gender				<0.001
Female	61.5	64.1	35.9	
Male	38.5	77.2	22.8	
Age group				<0.001
18-34	27.9	53.5	46.5	
35-49	28.8	68.2	31.8	
50-64	30.1	78.5	21.5	
65+	13.2	89.3	10.7	
Education				<0.001
High school or less	37.3	82.8	17.2	
Some college	35.7	66.8	33.2	
College graduate	17	56.9	43.1	
Post-graduate	10	54.6	45.4	
Household Income				<0.001
Less than $20,000	26.9	77.3	22.7	
$20,000 - $34,999	11.1	80.7	19.3	
$35,000 - $49,999	12.5	70.4	29.6	
$50,000 - $74,999	15.9	65.2	34.8	
$75,000 or more	33.5	59.6	40.4	
Race				0.96
White	70.6	69.9	30.1	
Black/ African American	9.6	68.4	31.6	
Hispanic	13.4	68.4	31.6	
Others	6.4	66.7	32.3	
Insurance status				0.62
No	8.3	66.3	33.7	
Yes	91.7	70	30	
Residence				0.7
Urban	87.3	69.4	30.6	
Rural	12.7	72	28	
Having a regular provider				0.79
No	28.6	70.3	29.7	
Yes	71.4	69.3	30.7	
Comorbidity				<0.001
None	43.7	62.2	37.8	
One	31.8	68	32	
At least 2 or more	24.5	84.1	15.9	
Body mass index (BMI)				0.02
Non-obese (BMI <30)	61.2	66.2	33.8	
Obese (BMI >= 30)	38.8	75.3	24.7	
Weekly physical activity				<0.001
<150 minutes	67.5	74	26	
>=150 minutes	32.5	61	39	
Weekly strength/ resistance training				0.03
<2 times/week	65.8	72.9	27.1	
>=2 times/week	34.2	63.7	36.3	

Wearable device use and physical activity levels

In the adjusted models (Table 2), WD use was not significantly associated with adherence to nationally recommended weekly levels of physical activity (OR 1.38, 95% CI (0.94, 2.04); p=0.10) or resistance strength training (OR 1.31, 95% CI (0.82, 2.08); p=0.26) among individuals with depression and/or anxiety.

**Table 2:** Multivariable logistic regressions for the association between wearable device use and physical activity levels among individuals with depression and anxiety OR - odds ratio, CI - confidence interval Analyses were adjusted for age, gender, race, educational level, household income, insurance status, comorbidities, access to a regular healthcare provider, body mass index, and geographical residence.

Outcomes	Unadjusted OR (95% CI)	p-value	Adjusted OR (95% CI)	p-value
Physical activity (>=150mins/week)	1.81 (1.628, 2.55)	0.01	1.38 (0.94, 2.04)	0.10
Strength training (>=2days/week)	1.53 (1.05, 2.23)	0.03	1.31 (0.82, 2.08)	0.26

In an additional exploratory analysis, we evaluated the association between the frequency of WD usage and similar physical activity outcomes. After adjusting for the same sociodemographic and health-related factors, we found that there was no significant difference in the odds of reporting adherence to nationally recommended weekly levels of physical activity (OR 1.37, 95% CI (0.71, 2.64); p=0.34) and resistance strength training (OR 1.24, 95% CI (0.68, 2.26); p=0.47) when comparing non-users to infrequent users. Similarly, there was no statistically significant difference in the odds of achieving nationally recommended weekly physical activity levels (OR 1.46, 95% CI (0.92, 2.33); p=0.11) or weekly resistance strength training (OR 1.42, 95% CI (0.84, 2.38); p=0.19) between non-users and frequent users of WD (Table 3).

**Table 3:** Multivariable logistic regressions for the association between frequency of wearable device use and physical activity levels among individuals with depression and anxiety OR - odds ratio, CI - confidence interval, WD - wearable device Analyses were adjusted for age, gender, race, educational level, household income, insurance status, comorbidities, access to a regular healthcare provider, body mass index, and geographical residence.

Variables	Unadjusted OR 95% CI	p-value	Adjusted OR 95% CI	p-value
Outcome: physical activity (>=150 mins/week)				
Reference (non-users)	1		1	
Infrequent users of WD	1.57 (0.89, 2.80)	0.12	1.37 (0.71, 2.64)	0.34
Frequent users of WD	2.00 (1.33, 3.08)	0.01	1.46 (0.92, 2.33)	0.11
Outcome: strength training (>=2 times/week)				
Reference (non-users)	1		1	
Infrequent users of WD	1.29 (0.75, 2.19)	0.35	1.24 (0.68, 2.26)	0.47
Frequent users of WD	1.73 (1.13, 2.65)	0.01	1.42 (0.84, 2.38)	0.19

## Discussion

The study aimed to explore the relationship between the utilization of wearable devices (WD) and physical activity engagement among individuals with mental disorders. Specifically, we characterized the frequency and prevalence of WD use, and examined the association between its use with adherence to nationally recommended guidelines for strength resistance exercise training and weekly physical activity. To conduct this investigation, data was collected from a national sample representing US adults diagnosed with depression and anxiety.

We found that roughly one in three adults with depression and/or anxiety reported use of WD in the 12 months preceding the survey, and among the WD users, about 70% reported frequent usage of these tools. In our study, amongst adults with depression and anxiety, the p-value for the gender variable is less than 0.001, indicating a significant association between gender and the use of wearable devices. Among the female participants, 35.9% of the total female respondents did use wearable devices, while among male participants, only 22.8% of the total respondents used them. However, 64.1% of the female respondents did not use wearable devices, while 77.2% of male respondents did not use them. The study also shows that the use of wearable devices is associated with a lower age group, with a p-value of less than 0.001. Among participants aged 18-34 years, 46.5% used wearable devices, while among participants aged 45-64 years, only 53.3% used them, and among participants aged 65 years and above, only 10.7% used them.

Our finding of 30% WD ownership among those with depression/anxiety matches estimates reported in the general adult population [26-28] and is also consistent with reports from past works [29]. Our results are encouraging and support the notion that not only has the digital divide narrowed among those with mental disorders but that these tools are feasible and readily acceptable for use in mental health contexts [15]. Thus, WD has potential and can serve as an added tool to support mental health delivery.

Beyond the continuous real-time monitoring and collection of passive data among people with mental disorders, increasing evidence suggests that WD may have benefits for health promotion and encouraging positive lifestyle change [15]. Despite the popularity of these tools in promoting physical activity, this study did not find an association between adherence to national recommended guidelines for weekly physical activities and the use of WD or strength/resistance exercises/training in people with depression/anxiety. Although previous studies suggest that WD may be linked with greater engagement in physical activity in the general population and other clinical populations [27,30], our results are contradictory to these findings but similar to other competing research [17], showing that perhaps the efficacy of these tools to promote physical activity in mental health contexts remains to be proven.

Multiple potential explanations exist for why the use of WD was not related to engagement in physical activity in our sample of US adults with depression and/or anxiety. First, individuals with depression and anxiety disorder may have different motivations and barriers to physical activity compared to the general population. For example, the intrinsic nature of depression and anxiety may lead to apathy, low self-esteem and reduce an individual's motivation to engage in physical activity. Past studies have found that intrinsic motivation remains a critical component of inducing and maintaining physical activity among people with mental disorders [31-33]. Second, people with depression and anxiety disorder may experience unique barriers to physical activity, such as fatigue and a lack of social and professional support, which may limit their ability to engage in physical activity even when using WD [34-36]. Third, another plausible explanation for the lack of association between WD use and physical engagement in our sample could be due to the limitations of the WD technology itself. It was unclear if these tools incorporated any elements of behavioral change theory, effective feedback with clinicians, or if they were customized to meet the individual's physical health needs, which are crucial elements for stimulating behavior change among those with mental disorders.

Nonetheless, WD provides a valuable opportunity for clinicians to continuously communicate, monitor and collect feedback data. Our findings suggest and emphasize that simply using these tools alone may not be sufficient to induce behavioral change in mentally ill populations. Rather, their potential and clinical utility in promoting actual engagement in physical activity in people with mental disorders may better be harnessed by incorporating users' perspectives, integrating elements of positive psychology and motivational behavioral constructs, and offering professional guidance and support with the use of these tools.

Limitations and strengths

Although our sample was nationally representative and weighted to obtain national estimates, it is important to interpret the findings of this study with the following limitations in mind. First, the cross-sectional nature of the study precludes causal conclusions between WD use and physical activity measures. Second, both WD use and physical activity measures were self-reported, which introduces the possibility of recall bias. Relatedly, we relied on self-reported diagnoses of depression and anxiety and were unable to validate depression/anxiety diagnosis with clinical data. Also, operationalizing the sample population "depression and anxiety" may have grouped individuals with a single entity into both. Our results are also not generalizable to patients with other mental health conditions, such as people with schizophrenia and bipolar disorder. Lastly, several useful factors, such as the duration of WD use and specific functionalities of the WD, were not captured due to the nature of the survey. Therefore, future research should consider employing longitudinal approaches, clinical records data, and objective measures for physical activity, such as accelerometers and pedometers to better comprehend the relationship between wearable technologies and physical activity in people with mental disorders.

## Conclusions

In conclusion, this study did not find a significant association between the use of WD and physical activities among people with anxiety and depression disorders. The findings suggest that in addition to using WD, those with depression and anxiety disorder may require different or customized interventions to increase physical activity levels compared to the general population. Future studies should examine how motivational and positive psychology behavioral constructs could be integrated with WD to support their role in physical health promotion in mental health contexts.
